# New Insights for the Design of Bionic Robots: Adaptive Motion Adjustment Strategies During Feline Landings

**DOI:** 10.3389/fvets.2022.836043

**Published:** 2022-04-21

**Authors:** Datao Xu, Huiyu Zhou, Xinyan Jiang, Shudong Li, Qiaolin Zhang, Julien S. Baker, Yaodong Gu

**Affiliations:** ^1^Faculty of Sports Science, Ningbo University, Ningbo, China; ^2^School of Health and Life Sciences, University of the West of Scotland, Paisley, United Kingdom; ^3^Department of Sport and Physical Education, Hong Kong Baptist University, Kowloon, Hong Kong SAR, China

**Keywords:** feline landing, bionic robots, deep learning method, finite element analysis, bionic engineering

## Abstract

Felines have significant advantages in terms of sports energy efficiency and flexibility compared with other animals, especially in terms of jumping and landing. The biomechanical characteristics of a feline (cat) landing from different heights can provide new insights into bionic robot design based on research results and the needs of bionic engineering. The purpose of this work was to investigate the adaptive motion adjustment strategy of the cat landing using a machine learning algorithm and finite element analysis (FEA). In a bionic robot, there are considerations in the design of the mechanical legs. (1) The coordination mechanism of each joint should be adjusted intelligently according to the force at the bottom of each mechanical leg. Specifically, with the increase in force at the bottom of the mechanical leg, the main joint bearing the impact load gradually shifts from the distal joint to the proximal joint; (2) the hardness of the materials located around the center of each joint of the bionic mechanical leg should be strengthened to increase service life; (3) the center of gravity of the robot should be lowered and the robot posture should be kept forward as far as possible to reduce machine wear and improve robot operational accuracy.

## Introduction

Animal species have inspired and helped to develop much of contemporary human technology. Without the inspiration obtained through animal models, the world's naturalistic progress would be impossible. Bionic robots are products that have developed and combined the characteristics of animals and human technology. Bionic robots' mobility mechanisms are often built on the bionics principle, replicating animal motion sections of the body or motion models while walking or running (1). Bionic robot design has gained momentum in recent years because robots have the potential to play a major role in replacing humans in difficult working environments, engage in rescue missions, space exploration, and so forth (2–5). Although wheeled and tracked robots move effectively on flat ground, the majority of bionic robots are capable of working in complicated and crowded terrain. Therefore, robots with legs can replicate humans and animals and are more adaptable for use in most situations. Previous studies have illustrated that the present legged robots include: a lizard bionic robot (6), a hexapod bionic robot (7), and an eight-legged bionic robot (8). It is worth mentioning that a leopard bionic robot devised by the Massachusetts Institute of Technology has a running speed that can reach 22 km/h (9). The previous studies used bionic mechanisms in the design of their robots, but their attention still focused on movement over flat ground. The benefits of bionic robots are that they are adapted to a variety of challenging terrains, such as debris rescue, geological exploration, and military reconnaissance. A robot incorporating jumping ability can jump to a level several times higher than its height (10); this function provides this jumping robot with excellent ability to move in complicated surroundings and compares well with the flea robot (11) and a miniature jumping robot (12).

The statement that it is easier to climb up the mountain than go down applies to this scenario. The design of the jumping robot did consider the ability for moving on complicated terrain but neglected to consider how the jumping robot returned to the initial jumping position. Quadrupeds have evolved a variety of distinct biological structures during the last million years, allowing them to adapt to a variety of habitats and terrains (13). As a typical quadruped, felines are well-known for their innate athletic abilities, particularly during jumping and landing (14, 15). Cats, because of their landing buffering mechanisms, may land safely from high locations without injuring themselves. When we consider the animal's capacity to land safely from great heights, the phrase “cats have nine lives” seems appropriate. Several examples have been documented in which the fatality rate of cats recorded when falling from great heights is <10% (16). Vnuk further went on to discover that when a feline fell from a great height, there was a 96.5% chance of survival. This intriguing phenomenon has initiated much scholarly curiosity. The cat can deal with the impact load of the ground easily. This can reduce joint injury and joint driving burden because of the cat's unique landing mechanism. The cats' forelimbs as the initial contact point for cats when performing a landing is one of the most important parts of the feline body during the landing phase. Previous studies have indicated that the forelimbs are important when performing a landing phase, as they absorb more weight, help with maneuvering, and are active during deceleration (17). Therefore, exploring the landing mechanism of each joint when a cat lands from different heights, provides new insights for the design of bionic robots.

Cats often jump from high levels, and their joints absorb several times their body weight in impact forces. Conventional biomechanical experiments (such as animal experiments, *in vitro* cadaveric specimens, etc.) often cannot fully reflect the real biomechanical changes of internal bones, but three-dimensional finite element analysis (FEA) can simulate the complex mechanical environment in a mathematical form and provide internal mechanical information (18, 19). FEA facilitates the measurement of external forces and the analysis of internal stresses during the experimental investigation and provides a better understanding of the cat's special landing mechanism. This knowledge has implications for the design of bionic robots, particularly during jumping and landing. In addition, a limiting factor in the construction of biomechanical models is the inability to analyze waveform data effectively, especially when different load factors affect the derived kinetic variables. Principal component analysis (PCA) is more sensitive than traditional parameter-based analysis techniques in detecting differences in kinematic and kinetic waveforms (20, 21). More and more studies are using PCA in time series datasets such as motion posture, gait, and ground reaction force (GRF), because PCA allows the detection of time-varying coordinated correlation patterns (22–25). Therefore, PCA can be used to extract the main characteristics of the GRF and motion posture of cats during landing, which can not only determine the potential relationship between variables but also reveal the main findings within the data set.

Recently, machine learning methods focusing on time series data analysis have been gradually applied in the field of motion analysis (such as support vector machine, artificial neural network, multivariable statistical analysis) (26–30). At the same time, the progress of motion capture technology, mechanical sensing technology, and signal processing technology makes biomechanical data acquisition diversified and refined, which provides the prerequisite for the application of big data-driven machine learning methods in the field of biomechanics (22, 28, 31). For example, artificial neural networks have been applied to gait pattern recognition and feature classification and realized personalized recognition and judgment of human gait patterns (27, 30, 32). Machine learning approaches have shown the potential to solve motion-related biomechanical problems and provide new insights into complex modeling systems. However, they all have the same problem of being a black box that does not provide any information about what makes the decisions (33, 34). The main reason is that all kinds of mappings in these models have non-linear characteristics, which leads to the lack of interpretability of classification prediction results (35). In the view of applications related to pattern recognition, the simple answers of “yes” or “no” sometimes have little or limited value because this does not validate classification decisions. Therefore, layer-wise relevance propagation (LRP) technology was proposed to solve the problem of lack of interpretability (35). LRP is a technology used to identify important relevance through backward propagation in neural networks, which measures the contribution of each input variable to the overall predicted outcomes. LRP has been successfully applied to many classification and recognition tasks in different scenarios, such as text, image, and pattern recognition (32, 36, 37). Therefore, the application of LRP in cat landing pattern recognition can improve the overall transparency of the classifier and make the classification results interpretable, thus providing reliable applied biomechanical diagnostic results.

Therefore, the purpose of this study was to investigate the biomechanical characteristics of a cat landing from different heights and provide new insights into bionic robot design based on the research findings and the needs of bionic engineering. According to previous studies, the segment parameters of the rigid body where the joints in the claw are far smaller than those of the rigid body where the wrist, elbow, and shoulder joints are (38), so this study only investigated the wrist, elbow, and shoulder joints in the inverse kinetics model. Specifically, this work was performed to investigate the adaptive motion adjustment strategy of the cat forelimb at each joint (wrist, elbow, and shoulder) during the landing phase using a machine learning algorithm and FEA. The first objective was to recognize and classify the kinematic and kinetic patterns of a cat's forelimbs when landing from different heights using the deep neural network (DNN) classification model and then to perform interpretability analysis of the classification results using LRP technology calculating the relevance score. The second objective was to reconstruct the waveform data (GRF and sagittal joint angle) during landing based on PCA, then extract the force and angle at the end of the landing phase (maximum elbow flexion) into the finite element model to analyze the stress distribution of the cat right forelimb bone. Finally, the aim of exploring the adaptive motion adjustment strategies of each joint during landing from different heights was achieved by combining the above results.

## Materials and Methods

Since the FEA can only investigate the stress distribution of the cat forelimb bone during the landing, and cannot discuss the biomechanical characteristics of the cat during the whole landing phase, this study combined the inverse kinetics model and the deep learning method to achieve the purpose of exploring the biomechanical characteristics of the whole landing phase of the cat, as well as the coordination strategy of each joint of the cat's forelimb when landing at different heights. Therefore, to more comprehensively explore the biomechanical characteristics of cat landing, the current study was mainly carried out from two aspects: (1) PCA and FEA; (2) inverse kinetics and deep learning method (DNN and LRP). Firstly, the GRF and joint kinematics (sagittal joint angle of wrist elbow shoulder) were collected when the cat landed from four different heights (60, 80, 100, and 120 cm). The landing phase was determined as the initial contact point to maximum elbow flexion. Then, the next steps are mainly divided into two steps. Step 1: Using PCA to reconstruct the data waveform of the three-direction GRF (anterior/posterior, medial/lateral, and vertical) and sagittal joint angle (wrist, elbow, and shoulder), the reconstructed waveform was the data waveform of the whole landing phase. Then, the GRF and joint angle data values of each landing height at the time point of the end of the landing phase (maximum elbow flexion) are extracted from the reconstructed waveform and substituted into the finite element model to investigate the stress distribution of the cat's right forelimb bone. Step 2: Three directions GRF (anterior/posterior, medial/lateral, and vertical) and sagittal joint angle (wrist, elbow, and shoulder) during the whole landing phase were taken as the imported data and then, the inverse kinetics to calculate the joint moment was used. After that, the data sets of each joint angle and moment during the whole landing phase were imported into the deep learning model to explore the landing strategies when cats land from different heights.

### Animals

A total of 60 healthy adult Chinese domesticated cats (aged 2.85 ± 0.49 years, body mass 4.32 ± 0.53 kg) were recruited *via* written consent from a local breeder for voluntary participation in this study. Prior to data collection, a full clinical examination was performed to ensure that there were no health issues that could impact the result of this study. Finally, a total of 56 subject cats were included in the experiment. A cat of moderate size, aged 3 years and weighing 4 kg, was selected from 56 cats. The cat was photographed by CT. The CT scan was obtained and performed by a qualified veterinarian in a pet hospital. Before obtaining the CT data, the cat was examined by a veterinarian to make sure there were no health problems or foot injuries. This study was approved by the Animal Care and Use Ethics Committee of Ningbo University (NBUAEC20200621).

### Experimental Protocol and Procedures

All tests were performed in the biomechanics laboratory at Ningbo University Research Academy of Grand Health. A force platform (Kistler, Switzerland) was set at a 1,000 Hz sampling frequency for GRF data collection when performing the landing task. Two high-speed cameras (Fastcam SA3, Photron, Japan) were set at 1,000 Hz and used for kinematic data collection during each landing task.

Before data collection, all cats were fully familiarized with the environment (test room), using toys and food to divert their attention. Before the formal start of the experiment, to ensure the smooth progress of the experiment, the cat was brought to the laboratory by its owner to tempt the cat with food or toys to complete the experimental process. This process lasted for 1 h each time, three times a week until the cat could be enticed by food and toys and could accurately jump to the designated area. The height of 0.6, 0.8, 1, and 1.2 m was taken as the heights selected for this experiment. Each cat was asked to jump from four heights, and 10 groups of data were collected for each height. A total of 40 groups of data were obtained from each one of the cats. To avoid cat fatigue, only one height was selected for 10 groups of data collection every test day.

The cat owner encouraged the cat to sit in a squat position on the jumping platform while the height of the table was adjusted to the specific required height. There was a 5-min break between each landing task to avoid inaccurate data collection caused by fatigue. There was no apparent tilt of the body, and the cat's head and body were facing forward when the cat landed. The experiment was considered successful when the cat's forelimb landed in the designated area and the cat continued to move away from the designated area, with no injuries or adverse reactions after the experiment.

**Figure 1:**
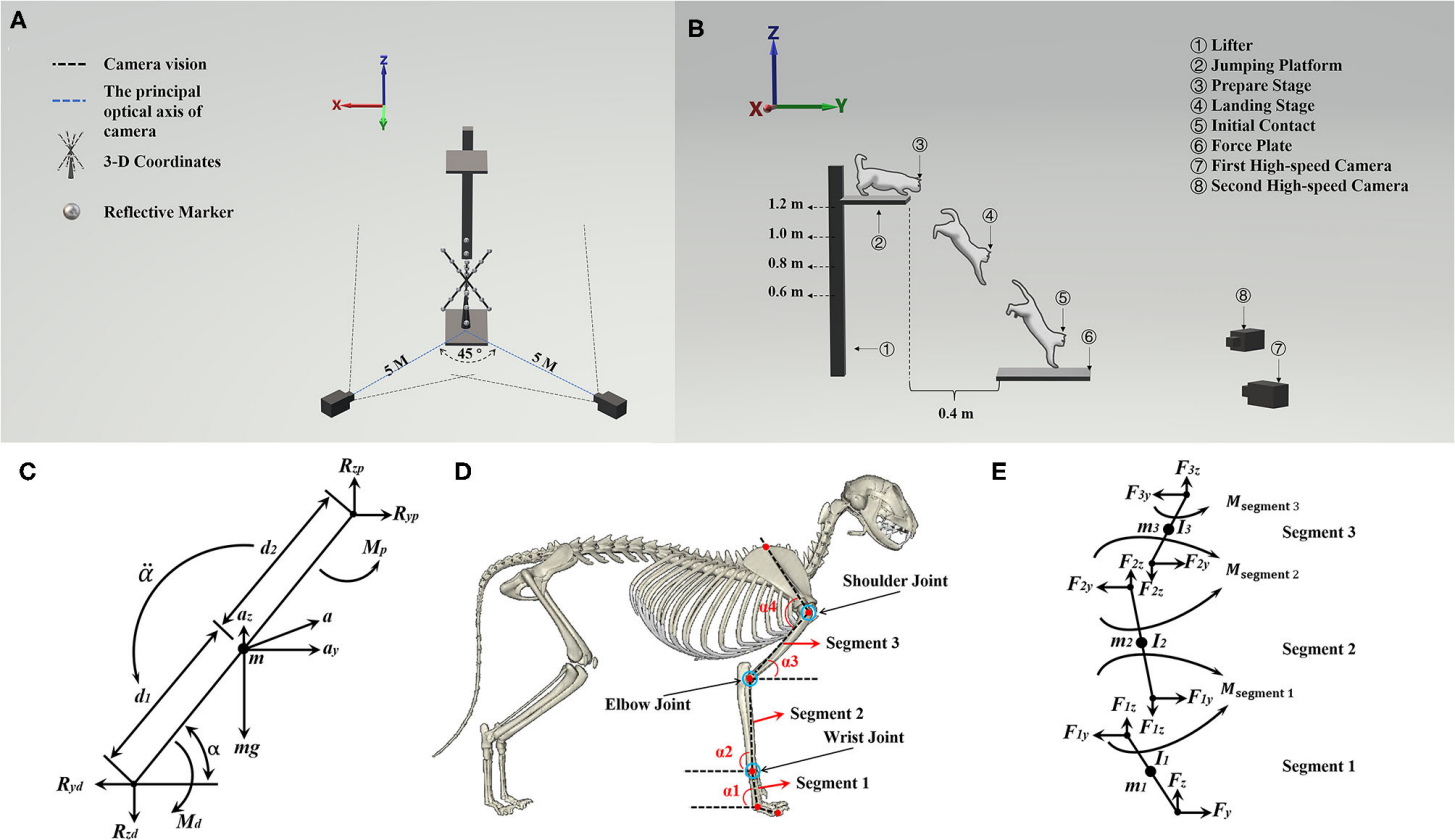
**(A)** Illustration of the position of two high-speed cameras and 3-D coordinates. **(B)** Illustration of cat landing procedure from the ready position to initial forelimbs contacting the ground; it also shows the position between the jumping platform and the force plate. **(C)** The complete free-body diagram of a single forelimb segment, shows reaction and gravitational forces, net moments of force, and all linear and angular accelerations. **(D)** Illustration of the position of red marker points on the forelimbs of cats (α_1_, α_2_, and α_3_ were used to calculate the joint moment, α_4_ is the shoulder joint angle), and the red marker point with a blue circle shows the bone marking the position of the wrist, elbow, and shoulder joint. **(E)** Freebody diagrams of the three rigid links, which include the joint reaction forces acting on each joint and the segment moment.

Figure 1A shows that two high-speed cameras were placed at the diagonal level of the force plate at a distance of 5 m from the landing target area, forming an ~45° angle between the principal optical axis of the two cameras. To build the space coordinate, three-dimensional (3-D) coordinates were placed on the center of the force platform. Figure 1B illustrates the cat landing from a ready position to initiate contact in the landing target area. Figure 1D shows the placement of a red marker point. The red marker point was used to ensure each one of the skeleton and joint positions for further data processing.

### Data Collection and Processing

The vertical GRF > 10 N was used to define the force plate's initial contact point (39). From the first contact point until the second peak vertical GRF time point, the landing phase was determined as the initial contact point (0% landing phase) to maximum elbow flexion (100% landing phase). Butterworth low-pass filters were used to filter the GRF data (filter order: fourth-order zero-phase lag, cut-off frequency: 50 Hz) (40). SIMI°-Motion 7.50 (Simi Reality Motion Systems GmbH, Munich, Germany) 3-D motion analysis system was used to analyze the landing phase of cats. Figure 1D shows the location of each trajectory marker point in red which was used for analysis. Then, the wrist, elbow, and shoulder sagittal plane joint angles were taken as an output from SIMI°-Motion, and the Butterworth fourth-order low-pass filter with a cutoff frequency of 6 Hz was used to digitally filter the original joint angle data. Each landing height (60, 80, 100, and 120 cm) of each joint (wrist, elbow, shoulder) of sagittal plane joint angle data and each landing height (60, 80, 100, and 120 cm) of each direction (X-axis: lateral and medial GRF; Y-axis: anterior and posterior GRF; Z-axis: vertical GRF) of GRF data were expanded into 100 data point's curve by a self-written MATLAB script. Finally, the dataset of GRF and joint angle were input to MATLAB to run the Inverse Kinetics Algorithm and PCA.

### Inverse Kinetics Algorithm

In this study, we investigated only the sagittal motion of cat landing, as the main motion of a cat on landing is in the sagittal plane (41, 42). Therefore, the GRF and joint angle of the wrist, elbow, and shoulder during the landing phase were taken as the imported data, and then the inverse kinetics algorithm to calculate the joint moment of the wrist, elbow, and shoulder was used. The Y-axis was defined as the anterior/posterior direction, and the Z-axis was defined as the vertical direction. The right forelimb of the cat was analyzed by splitting into three rigid links and considered as a rigid body model of planar link segment (arm: segment 1; forearm: segment 2; carpals: segment 3). At the same time, the segment parameters (segment mass, moment of inertia) were obtained based on the previous study (38), which combined the joint kinematics and GRF to calculate the joint moment based on the inverse kinetics (43). Each forelimb segment was assumed to act separately under a combination of gravity, joint reaction forces, and muscle moments. As shown in Figure 1C, *Eq*. 1, *Eq*. 2, and *Eq*. 3 can be obtained based on Figure 1C (43):


(1) $$\begin{array}{l} \sum F_{y}=ma_{y}=R_{yp}-R_{yd} \end{array}$$

(2) $$\begin{array}{l} \sum F_{z}=ma_{z}=R_{zp}-R_{zd}-mg \end{array}$$

(3) $$\begin{array}{l} \sum M=M_{p}-M_{d}=I_{0}\ddot{\alpha } \end{array}$$

where the *F*_*y*_ is the reaction forces in the Y-axis direction and the *F*_*z*_ is the reaction forces in the Z-axis direction; the *m* is the mass of segment; the *a*_*y*_ and *a*_*z*_ are the Y-axis and Z-axis components of acceleration of the center of mass (COM), respectively; *M* is the joint moment of the current segment; *M*_*d*_ and *M*_*p*_ are the distal and proximal joint moment of the segment, respectively; *I*_0_ is the moment of inertia in the plane of movement; α is the angle of the segment in the plane of movement; $\ddot{\alpha }$ is the angular acceleration of the segment, and the arrow below the $\ddot{\alpha }$ is the direction of the $\ddot{\alpha }$. In Figure 1C, the *R*_*yp*_ and *R*_*zp*_ are the Y-axis and Z-axis direction proximal joint reaction force, and the *R*_*yd*_ and *R*_*zd*_ are the Y-axis and Z-axis direction distal joint reaction force.

The right forelimb of the cat was analyzed by splitting the forelimb into three rigid links (as shown in Figures 1D,E). The COM was set at the midpoint of each segment. Then, Eq. 4, Eq. 5, and Eq. 6 can be derived from Eq. 1, Eq. 2, and Eq. 3. Therefore, the joint moment in segment 2 (Eq. 7, Eq. 8, and Eq. 9) of the right forelimb can be calculated according to segment 1 (Eq. 4, Eq. 5, and Eq. 6), and segment 3 (Eq. 10, Eq. 11, and Eq. 12) of the right forelimb can be calculated according to segment 2 (Eq. 7, Eq. 8, and Eq. 9).

For segment 1:


(4) $$\begin{array}{l} F_{1y}-F_{y}=m_{1}\left(-\frac{L_{1}}{2}{\dot{\alpha }}_{1}^{2}\cos\alpha_{1}-\frac{L_{1}}{2}{\ddot{\alpha }}_{1}\sin\alpha_{1}\right) \end{array}$$

(5) $$\begin{array}{l} F_{1z}-F_{z}-m_{1}g=m_{1}\left(-\frac{L_{1}}{2}{\dot{\alpha }}_{1}^{2}\sin\alpha_{1}+\frac{L_{1}}{2}{\ddot{\alpha }}_{1}\cos\alpha_{1}\right) \end{array}$$

(6) $$\begin{array}{l} M_{\text{segment }1}+F_{1y}\frac{L_{1}}{2}\sin\alpha_{1}-F_{1z}\frac{L_{1}}{2}\cos\alpha_{1}+F_{y}\frac{L_{1}}{2}\sin\alpha_{1}\\ \;-F_{z}\frac{L_{1}}{2}\cos\alpha_{1}=-I_{1}{\ddot{\alpha }}_{1} \end{array}$$

For segment 2:


(7) $$\begin{array}{l} F_{2y}-F_{1y}=m_{2}[-L_{1}\left({\dot{\alpha }}_{1}^{2}\cos\alpha_{1}+{\ddot{\alpha }}_{1}\sin\alpha_{1}\right)\\ \;-\frac{L_{2}}{2}\left({\dot{\alpha }}_{2}^{2}\cos\alpha_{2}+{\ddot{\alpha }}_{2}\sin\alpha_{2}\right)] \end{array}$$

(8) $$\begin{array}{l} F_{2z}-F_{1z}-m_{2}g=m_{2}[L_{1}\left(-{\dot{\alpha }}_{1}^{2}\sin\alpha_{1}+{\ddot{\alpha }}_{1}\cos\alpha_{1}\right)\\ \;+\frac{L_{2}}{2}(-{\dot{\alpha }}_{2}^{2}\sin\alpha_{2}+{\ddot{\alpha }}_{2}\cos\alpha_{2})] \end{array}$$

(9) $$\begin{array}{l} M_{\text{segment }2}-M_{\text{segment }1}+F_{2y}\frac{L_{2}}{2}\sin\alpha_{2}-F_{2z}\frac{L_{2}}{2}\cos\alpha_{2}\\ \;+F_{1y}\frac{L_{2}}{2}\sin\alpha_{2}-F_{1z}\frac{L_{2}}{2}\cos\alpha_{2}=-I_{2}{\ddot{\alpha }}_{2} \end{array}$$

For segment 3:


(10) $$\begin{array}{l} F_{3y}-F_{2y}=m_{3}[-L_{1}\left({\dot{\alpha }}_{1}^{2}\cos\alpha_{1}+{\ddot{\alpha }}_{1}\sin\alpha_{1}\right)\\ \;-L_{2}\left({\dot{\alpha }}_{2}^{2}\cos\alpha_{2}+{\ddot{\alpha }}_{2}\sin\alpha_{2}\right)\\ \;+\frac{L_{3}}{2}({\dot{\alpha }}_{3}^{2}\cos\alpha_{3}+{\ddot{\alpha }}_{3}\sin\alpha_{3})] \end{array}$$

(11) $$\begin{array}{l} F_{3z}-F_{2z}-m_{3}g=m_{3}[L_{1}\left(-{\dot{\alpha }}_{1}^{2}\sin\alpha_{1}+{\ddot{\alpha }}_{1}\cos\alpha_{1}\right)\\ \;+L_{2}(-{\dot{\alpha }}_{2}^{2}\sin\alpha_{2}+{\ddot{\alpha }}_{2}\cos\alpha_{2})\\ \;+\frac{L_{3}}{2}(-{\dot{\alpha }}_{3}^{2}\sin\alpha_{3}+{\ddot{\alpha }}_{3}\cos\alpha_{3})] \end{array}$$

(12) $$\begin{array}{l} M_{\text{segment }3}-M_{\text{segment }2}+F_{3y}\frac{L_{3}}{2}\sin\alpha_{3}-F_{3z}\frac{L_{3}}{2}\cos\alpha_{3}\\ \;+F_{2y}\frac{L_{3}}{2}\sin\alpha_{3}-F_{2z}\frac{L_{3}}{2}\cos\alpha_{3}=I_{3}{\ddot{\alpha }}_{3} \end{array}$$

where the *F*_1*y*_, *F*_2*y*_, and *F*_3*y*_ are the Y-axis direction proximal joint reaction force of segment 1, segment 2, and segment 3, respectively; the α_1_, α_2_, and α_3_ are the angles of segment 1, segment 2, and segment 3 with the horizontal plane, respectively; the α_4_ is the shoulder joint angle; the *F*_1*z*_, *F*_2*z*_, and *F*_3*z*_ are the Z-axis direction proximal joint reaction force of segment 1, segment 2, and segment 3, respectively; *m*_1_, *m*_2_, and *m*_3_ are the mass of segment 1, segment 2, and segment 3, respectively; the *L*_1_, *L*_2_, and *L*_3_ are the length of segment 1, segment 2, and segment 3, respectively; the *M*_segment 1_, *M*_segment 2_, and *M*_segment 3_ are the proximal joint moment of segment 1, segment 2, and segment 3, respectively; the *I*_1_, *I*_2_, and *I*_3_ are the moment of inertia of segment 1, segment 2, and segment 3, respectively. α_1_, α_2_, α_3_, and α_4_ were the angle defined by this study, which was collected by the high-speed camera and mainly used to calculate the joint moment. According to a previous study (44), the mass of the arm, forearm, and carpals is 2.37, 1.30, and 0.30% of the body mass, respectively. The moment of inertia at COM of the arm, forearm, and carpals are 391.81, 233.34, 7.51, respectively (unit: g^*^cm^2^).

The whole inverse kinetics algorithm was realized by a self-written MATLAB (MATLAB R2019a, MathWorks, United States) script. Then, the joint moment of the wrist, elbow, and shoulder was obtained by inverse kinetics. Each landing height (60, 80, 100, and 120 cm) of each joint (wrist, elbow, and shoulder) of sagittal plane joint kinematics (joint angle) and joint kinetics (joint moment) data were expanded into 100 data point's curve by a self-written MATLAB script too. Finally, four matrices were obtained (representing four datasets from different landing heights): *M*_60 cm_, *M*_80 cm_, *M*_100 cm_, *M*_120 cm_. Where the *M*_60 cm_, *M*_80 cm_, *M*_100 cm_, and *M*_120 cm_ are the data matrices (including the data sets of joint angle and joint moment) of 60 cm, 80 cm, 100 cm, and 120 cm landing height, respectively. The dimensions of these four matrices are all $560_{\text{row}}^{*}600_{\text{column}}$, 560 represents 560 successful trails (a total of 56 subject cats and 10 successful data were collected for each cat), and 600 represents 600 time-series data points (3 sets of kinematic data and 3 sets of kinetic data, each of which contains 100 data points). The *M*_1_ (*M*_1_ = *M*_60 cm_ + *M*_80 cm_), *M*_2_ (*M*_2_ = *M*_80 cm_ + *M*_100 cm_), and *M*_3_ (*M*_3_ = *M*_100 cm_ + *M*_120 cm_) are the data matrices that combined the *M*_60 cm_ and *M*_80 *cm*_, *M*_80 cm_ and *M*_100 cm_, *M*_100 cm_ and *M*_120 cm_, respectively. Finally, a total of three times DNN and LRP analyses were performed, which included input matrices *M*_1_, *M*_2_, and *M*_3_ independently.

### Data Analysis

#### Principal Component Analysis

The PCA is a multivariate statistical analysis method that converts multiple indexes into several comprehensive indexes by orthogonal rotation transformation with the idea of dimensionality reduction and the premise of losing little information. The comprehensive index generated by transformation is usually called the principal component (PC), in which each PC is a linear combination of the original variable, and each PC is unrelated to the other. It is also an unbiased method for extracting relevant information from high-dimensional data, considering the major components that account for a large portion of the total data set. In this way, it is possible to consider only a few principal components without losing too much information when studying complex problems. Therefore, it is easier to grasp the main contradiction, reveal the regularity between the internal variables of things, and simplify the problem to improve the efficiency of analysis (45–47). The primary function of PCA is to obtain a set of non-redundant variables to describe a certain phenomenon or process compactly (data dimension reduction). In other words, the reconstruction of waveform data using PCA can extract the main features of the waveform and determine the underlying relationships between variables. Therefore, the waveform data reconstructed by PCA can represent the most important part of the whole data set, rather than the simple average value. From a numerical point of view, some of the waveform data of landing at different heights vary greatly, while others vary little. This goes back to the nature of the data features, and what represents the nature of the current data set is the data that has changed a lot or a little after reconstruction. Therefore, PCA was used to reconstruct the data waveform in this study.

In this work, the dataset of GRF and joint angle were conducted by PCA, the waveform data of each variable was then reconstructed. For the dataset of GRF, separate PCA was conducted for each height (0.6, 0.8, 1, 1.2 m) in each direction (X-axis: lateral and medial GRF; Y-axis: anterior and posterior GRF; Z-axis: vertical GRF) resulting in twelve analyses direction. For the dataset of joint angle, separate PCA was conducted for each height (0.6, 0.8, 1, 1.2 m) in each joint (wrist joint, elbow joint, shoulder joint) resulting in twelve analyses direction. For each PCA, a total of 560 sets of data were designed, and each set corresponded to 100 data points, combined into a 100 × 560 (*n* × *p*) matrix. The 560-dimensional vector constituted by these 560 groups of data is the original variable *X*_1_


$$X_{1}=\;\left[\begin{array}{cccc} x_{11}\; & x_{12}\; & \cdots \; & x_{1p}\\ x_{n1}\; & x_{22}\; & \cdots \; & x_{2p}\\ \vdots \; & \ddots \; & \ddots \; & \vdots \\ x_{n1}\; & x_{n2}\; & \cdots \; & x_{np} \end{array}\right]=\left(x_{1},x_{2},{\ldots ,x}_{p}\right)$$

where n represents 100 data points after interpolation and using *t*(*i*) (*i* = 1, 2, …, *n*) to denote the specific time point in the landing phase. At each specific time point, the cat had a specific landing posture and corresponded to a specific vector in the GRF and joint angle. The matrix *X*_1_ was normalized by the “zscore” function of MATLAB, and this function was based on the mean and standard deviation of the original data for data normalization. After that, the covariance matrix *Cov*(*X*_1_) was calculated based on the normalized matrix. The eigenvalues λ_*i*_ were extracted from the covariance matrix *Cov*(*X*_1_), as well as orthogonalized unit eigenvectors β_*i*_ were calculated from the covariance matrix *Cov*(*X*_1_). The eigenvalues λ_*i*_ following ranking λ_1_ ≥ λ_2_ ≥ … ≥ λ_*p*_ ≥ 0 with $\overset{p}{\underset{i=1}{\sum }}\lambda_{i}=1$. The orthogonalized unit eigenvectors β_*i*_ is the coefficient of PC scores *PC*_*i*_ concerning the original variable *X*_1_. The PC scores *PC*_*i*_ represent important landing waveform characteristics, which include overall magnitude, timing differences, and shape (the differences in the amplitude during different time points or phases). The principal component(*PC*_1_, *PC*_2_, …, *PC*_*m*_) to be selected is determined by the accumulative contribution rate of variance information *G*(*m*), and it was calculated as


(13) $$\begin{array}{l} G\left(m\right)=\frac{\sum_{i=1}^{m}\lambda_{i}}{\sum_{k=1}^{p}\lambda_{k}} \end{array}$$

According to the values of the accumulative contribution rate of variance information *G*(*m*), the number of PC scores *PC*_*i*_ were determined. Finally, the waveform data were reconstructed based on the PC scores, that is also called the principal GRF and principal joint angle. Specifically, the selected PC scores were multiplied by the transpose of the PC coefficient matrix. Then, each sample was multiplied by the sample's standard deviation vector with the addition of the mean vector to reconstruct waveforms (30). The GRF (anterior/posterior, medial/lateral, vertical) value and sagittal joint angle (wrist, elbow, shoulder) value at the time point of maximum elbow flexion during the landing phase were extracted from the reconstructed waveform (principal GRF and principal joint angle) then imported into Mimics to FEA, respectively.

#### Finite Element Analysis

**Figure 2:**
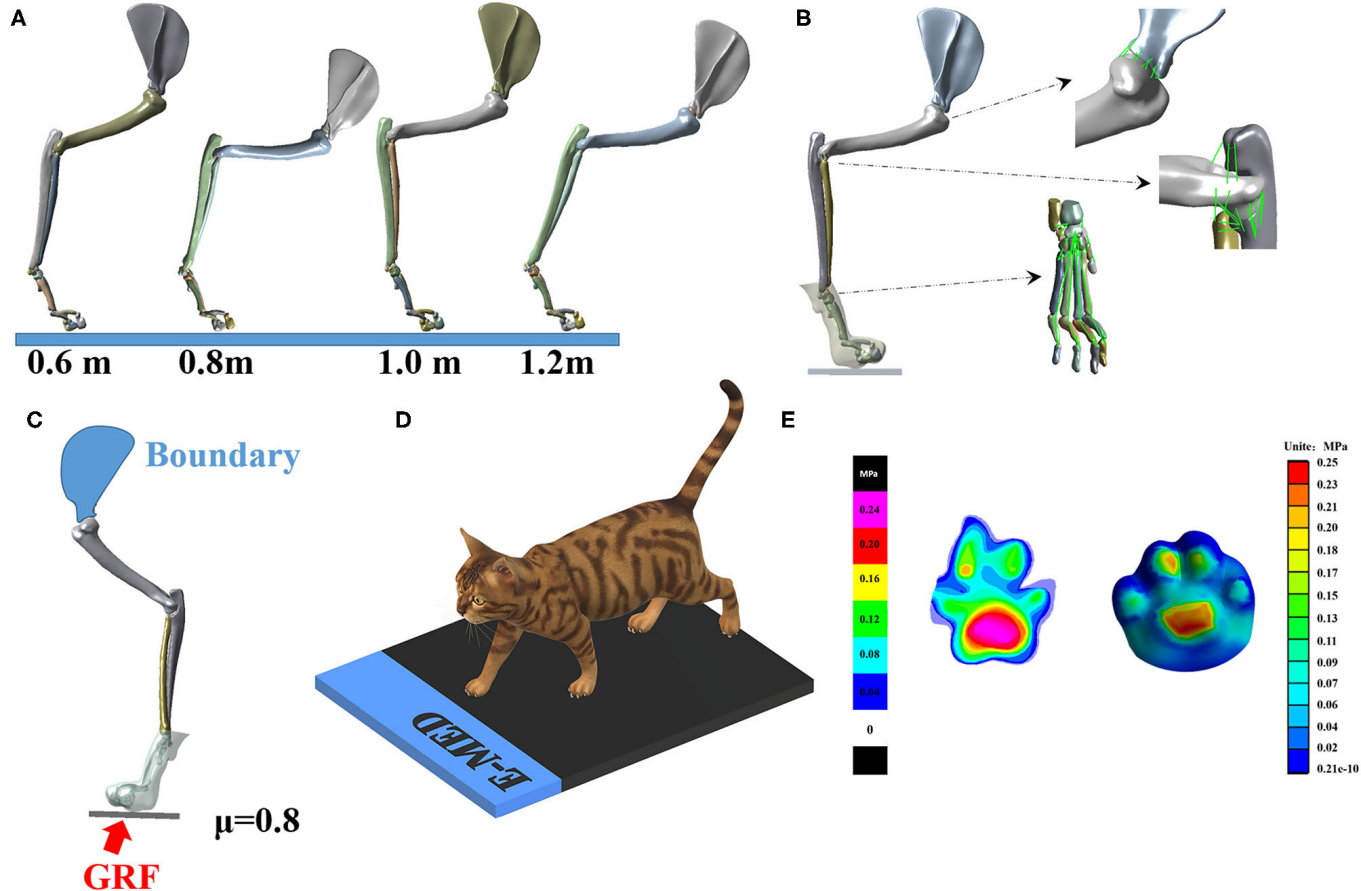
**(A)** Finite element 3D model of right forelimb at four landing heights. **(B)** Illustration of ligament and soft tissue of model. **(C)** Illustration of loading and boundary conditions. **(D)** Experimental verification of plantar pressure of cat right forelimbs. **(E)** Experimental verification results (left) and finite element simulation results (right). The scales on the left and right diagrams are different; please refer to their scales.

The whole-body CT images of cats were collected at 0.5 mm intervals, and only the right forelimb was analyzed. Previous studies have shown that the maximum elbow flexion point during landing is a turning point that best represents the characteristics of cat landing patterns (42, 48). Therefore, this study established a finite element model based on the position of each bone joint during maximum elbow flexion. MIMICS16.0 (Materialise, Leuven, Belgium) segmented 25 bones (one scapula, one humerus, one radius, one ulna, seven carpal bones, five metacarpals, and nine phalanges) and their inclusions. The output of each bone was placed in STL format and imported into Geomagic (Geomagic, Inc., Research Triangle Park, NC, United States) for smoothing. Finally, the IGES file format for each bone was exported. The model in SolidWorks (SolidWorks Corporation, MA, United States, 2017) was assembled, according to the corresponding angles of different falling heights, establishing a three-dimensional solid model and a verification model of cat forelimbs under four different heights (the joint angle of each joint of each landing height are shown in Figure 2B), and generating ligaments according to anatomical characteristics (49). The final model is shown in Figures 2A,B.

The cat's lower limbs and related parts have similar structures, so all tissues are idealized as linear elastic isotropic materials. According to the published literature (50–52). The material properties of each part are shown in Table 1. The soft tissue and bone in the finite element model are modeled based on CT images, and the cartilage is modeled in Solidworks based on the anatomical structure of the cat's forelimb. All of the above are solid parts. Ligaments are simulated in the Workbench (ANSYS, Inc., Canonsburg, United States) using line elements that only stretch. The modeling of the above elements is strictly based on the anatomical structure of the cat's forelimb.

**Table 1:** Material properties of the cat's right forelimbs model components.

**Component**	**Young's modulus E (MPa)**	**Poisson's ratio ν**
Paw	0.15	0.45
Bone	15,000	0.3
Cartilage	1	0.4
Ligaments	260	0.4
Plate	17,000	0.4

The purpose of FEA was to investigate the stress distribution of the cat's right forelimb bone when the cat is hit by an external force. In this study, the PCA was used to optimize the measured GRF, and the reconstructed GRF waveform data is the external force borne by the cat when it lands from the height plate, so the external force was used as the input of the finite element model. The right forelimb model under static conditions is considered. Load and boundary conditions are applied to the right forelimb of the cat. The boundary condition is fixed on the inside of the scapula, and the load is applied to the lower surface of the ground to simulate the external force when falling. The medial edge of the scapula was fixed. The interaction between the foot and the ground is simulated as a foot system, which is a commonly used method in biomechanical modeling of the human foot (Figure 2C). The plate is endowed with elastic properties to simulate concrete ground support. The vertical external force is applied under the plate, and the external force of different heights is shown in Figure 2D. This study adopts two kinds of contact relations: (1) binding, there is no relative sliding displacement between the two parts of the binding under force; (2) friction, the contact condition between soft tissue and bone is set as binding, and the claw produces friction contact (μ = 0.8). At the same time, due to the presence of synovial fluid, the friction coefficient between bone and cartilage at the joint was determined to be 0.01(μ = 0.01).

The verification of the model refers to the human foot numerical model, and the finite element foot model is verified by the plantar pressure distribution (53–55). Therefore, the paw contact pressure and contact area distribution of cats are also extracted from the pressure platform measurements (Munich novelty, Germany). The experimental pressure data are collected under static standing conditions, and the cat is placed quietly on the force platform with a GRF of 1/4 of body weight for comparison with simulation results (52) (Figure 2D).

#### DNN Model and Layer-Wise Relevance Propagation Analysis

Neural networks are extensive parallel networks composed of adaptive simple units whose organization can simulate the interactions of biological nervous systems to real-world objects (56). Neural networks with more than two hidden layers are defined as DNNs. It is generally believed that the DNN can improve the accuracy of the whole model (57). In this study, the application of the DNN model was mainly biased to improve the accuracy of the model (57). In other applications of neural networks, the reason they don't use DNN is that DNN is less efficient (time-consuming), which in many cases is not allowed. However, the current study does not consider the operation efficiency, so a DNN model with 10 hidden layers was designed under the condition of repeated model training and adjustment according to the actual data. The matrices *M*_1_, *M*_2_, and *M*_3_ were conducted using LRP analysis respectively. For the input matrices *M*_1_, the data of the *M*_60 *cm*_ was set at positive class, and the data of the *M*_80 *cm*_ was set at negative class. For the input matrices *M*_2_, the data of the *M*_80 *cm*_ was set at positive class, and the data of the *M*_100 *cm*_ was set at negative class. For the input matrices *M*_3_, the data of the *M*_100 *cm*_ was set at positive class, and the data of the *M*_120 *cm*_ was set at negative class. Before the data training, 1,120 sample data sets were randomly distributed through the functions, and then 80% of the data sets (896 sample data sets) were extracted as training sets, and the remaining 20% (224 sample data sets) as test sets.

First, a DNN was established that included one input layer, 10 hidden layers, and one output layer, and the per-layer nodes were determined by the input data shape (27). Therefore, the nodes of the input layer, hidden layers, and output layer were 600, 1,200, and 2. The layers of the neural network are fully connected, which means the neuron of the *n*-th layers must be connected to the neuron of the (*n* + 1)-th layer. A linear relation function and an activation function were used to calculate the new values between layers. The linear relationship function *z* of the model constructed in this study is:


(14) $$\begin{array}{l} z=\;\overset{n+1-th}{\underset{i=1}{\sum }}w_{i}u_{i}+b \end{array}$$

where *w*_*i*_ is the connection weight of the *i*-th neuron, the *u*_*i*_ is the input from the *i*-th neuro, the *b* is the constant of the function, and the *m*−*th* is the *m*−*th* layers neural network. The activation function *A*(*c*) of the hidden layer used the hyperbolic tangent function:


(15) $$\begin{array}{l} A\left(c\right)=\;\frac{e^{c}-e^{-c}}{e^{c}+e^{-c}} \end{array}$$

where the *c* is the input scalar of the hyperbolic tangent function. The hyperbolic tangent function expands the mapped range of the Sigmoid function from [0, 1] to [-1, 1], which produces better training performance. The batch size was set at 25, and the epoch limit was set at 3,000. Following DNN training, the relevance score was calculated by the LRP, and the performance of the classifier was evaluated by the accuracy achieved and other parameters.

Layer-wise relevance propagation is a technology used to identify important relevance through backward propagation in neural networks. Backward propagation is a conservative relevance redistribution process in which the neurons that contribute the most to the upper layer receive the most relevance from the upper layer. In general, LRP aims to narrow the gap between the classification and interpretability of multi-layer neural networks on non-linear cores (35, 58, 59).

The overall idea is to understand the contribution of a single feature of dataset *x* to the prediction *f*(*x*) made by the classifier *f* in pattern recognition and classification tasks. That is, the positive or negative contribution of each feature to the classification result for dataset *x* can be calculated, and the degree of such contribution can be accurately measured to a certain extent (The contribution of each input feature *x*(*d*) to a particular prediction *f*(*x*); the *d* is the input data of *x*(*d*) function). In the setting of the classifier is a mapping *f*: *J*^*v*^ → *J*^1^ (*J* is a generic symbol for mapping; *v* is the *v*-th layers), *f*(*x*)>0 indicates the existence of a learning structure. The constraint of classification is to find the differential contribution relative to the most uncertain state of the classification, which is then represented by the root point *f*(*x*_0_) = 0. By factoring the prediction *f*(*x*) into the sum of the individual input feature *x*(*d*):


(16) $$\begin{array}{l} f\left(x\right)=\;\overset{v}{\underset{d=1}{\sum }}R_{d} \end{array}$$

where *R*_*d*_ is the relevance score of the *d*-th layers. In the classifier, whether for non-linear support vector machines or neural networks, the first layer is the input features, and the last layer is the predicted output of the classifier. Meanwhile, each layer is part of the features extracted from dataset *x* after running the classification algorithm. The *l*-th layer is modeled as a vector ${z=\left(z_{d}^{l}\right)}_{d=1}^{V\left(l\right)}$ with dimensionality *V*(*l*). LRP has a relevance score $R_{d}^{\left(l+1\right)}$ for each dimension $z_{d}^{\left(l+1\right)}$ of vector *z* at layer *l*+1. A relevance score $R_{d}^{\left(l\right)}$ is found in each dimension $z_{d}^{l}$ of vector *z* near the next layer *l* of the input layer, as shown in the following formula:


(17) $$\begin{array}{l} f\left(x\right)=\ldots =\underset{d\in l+1}{\sum }R_{d}^{\left(l+1\right)}=\underset{d\in l}{\sum }R_{d}^{\left(l\right)}=\ldots =\underset{d}{\sum }R_{d}^{\left(1\right)} \end{array}$$

The inter-hierarchical relevance is represented by the message $R_{i\leftarrow j}^{\left(l,\;l+1\right)}$ between neuron *i* and *j*, and these messages can be sent along with each connection. The output *f*(*x*) is then passed from one neuron to the next by backward propagation. The relevance of neurons is defined as the sum of incoming messages; then the sum runs over the sinks at layer *l*+1 for a fixed neuron *i* at layer *l*.


(18) $$\begin{array}{l} R_{j}^{\left(l\right)}=\underset{k:\;i\;\text{is input for neuron }j}{\sum }R_{i\leftarrow j}^{\left(l,\;l+1\right)} \end{array}$$

The input of the next neuron in the direction is defined during classification; then the sum runs over the sources at layer *l* for a fixed neuron *k* at layer *l*+1. In general, this can be expressed as:


(19) $$\begin{array}{l} R_{k}^{\left(l+1\right)}=\underset{i:\;i\;is\;input\;for\;neuron\;k}{\sum }R_{i\leftarrow k}^{\left(l,\;l+1\right)} \end{array}$$

The relevance of each layer is calculated by backward propagation: the relevance $R_{i}^{\left(l\right)}$ is expressed as a function of the upper relevance $R_{j}^{\left(l+1\right)}$, and the back propagates the relevance until the input feature is reached. By the relevance of the neuron $R_{j}^{\left(l+1\right)}$to the classification decision *f*(*x*), the relevance is then decomposed according to the message *R*_*i*←*j*_ sent to the upper layer of neurons. Holding the conservation property:


(20) $$\begin{array}{l} \underset{i}{\sum }R_{i\leftarrow j}^{\left(l,\;l+1\right)}\;{=R}_{j}^{\left(l+1\right)} \end{array}$$

For the linear network $f\left(x\right)=\underset{i}{\sum }z_{ij}$, the relevance is *R*_*j*_ = *f*(*x*), and the decomposition is directly by *R*_*i*←*j*_ = *z*_*ij*_. Through hyperbolic tangent function and rectification function two monotone increasing functions, the pre-activation function *z*_*ij*_ provides a reasonable way to measure the relative contribution of *x*_*i*_ to *R*_*j*_ for each neuron. Based on the proportion of local pre-activation and global pre-activation, the selection of association decomposition is obtained:


(21) $$\begin{array}{l} R_{i\leftarrow j}^{\left(l,\;l+1\right)}=\frac{z_{ij}}{z_{j}}*R_{j}^{\left(l+1\right)} \end{array}$$

where *z*_*j*_ is the weight connecting the neuron *x*_*j*_. The relevance *R*_*i*←*j*_ are shown in


(22) $$\sum_{i}R_{i\leftarrow j}^{\left(l,\;l+1\right)}\;=R_{j}^{\left(l+1\right)}*\left(1-\frac{b_{j}}{z_{j}}\right)$$

where *b*_*j*_ is the bias term of the *j*-layer neuron. Multiplier accounts represent the relevance absorbed by the bias term, and the residual bias correlations can be reassigned to each neuron *x*_*i*_. According to the determined rule (*Eq*. 21), through adding up the correlations of all neurons in the upper layer *i* (combined *Eq*. 18 and *Eq*. 19), the overall relevance of all neurons in the next layer *j* can be obtained:


(23) $$\begin{array}{l} R_{i}^{\left(l\right)}=\underset{j}{\sum }R_{i\leftarrow j}^{\left(l,\;l+1\right)} \end{array}$$

The relevance propagates from one layer to another until it reaches the input feature *x*(*d*), where the relevance $R_{d}^{\left(1\right)}$ provides the hierarchical eigen-decomposition required for the decision *f*(*x*). More details can be found by referring to Sebastian's study (58). All algorithms were run in MATLAB R2019a, by self-written scripts according to the LRP toolbox (60).

The relevance of correctly classified cat landing patterns was extracted by defining logical variables, and then a relevance score was assigned to each input variable. LRP determines the correlation between each variable and the predicted results of the model and normalizes the LRP-derived association patterns to their respective maximum values for comparison. Since the input variables are collected in the time domain, and the adjacent values are interdependent, the fluctuation of the relevance score can be reduced by smoothing. Therefore, the average of the correlation patterns was corrected and smoothed; then the smoothed correlation pattern was rescaled from 0 (no correlation) to 1 (the highest correlation). The whole smoothing process was repeated three times, with the preceding and following points of each point weighted by 25%. The sum of weights equal to 1, which was accomplished by simulating the Gaussian filter. To explore the influence of different variables on the accuracy of model classification, all variables were sorted according to the correlation between variables, and then the top 100 variables with the highest relevance scores were selected to explain and analyze the cat landing pattern.

#### Evaluate the Performance of the Classifier

Combine the results of the classification model into a 2^*^2 table called confusion matrix $m=\left(\begin{array}{cc} TP\; & FN\\ FP\; & TN \end{array}\right)$, which fully describes the results of the classification task (61). True Positives (TP): Actual positives that are correctly predicted positives; False Negatives (FN): Actual positives that are wrongly predicted negatives; True Negatives (TN): Actual negatives that are correctly predicted; False Positives (FP): Actual negatives that are wrongly predicted positives.

Then, considering the possibility of unbalanced class distribution, the following indicators were calculated to evaluate the performance of the classifier.

1. The accuracy of a classifier on a given set of tests is the percentage of tuples that are correctly classified by the classifier:
  (24) $$\begin{array}{l} \text{accuracy}=\;\frac{TP+TN}{TP+FN+FP+TN} \end{array}$$
2. The sensitivity (also called recall) is the true positive cases recognition rate, which means the percentage of positive tuples correctly identified:
  (25) $$\begin{array}{l} \text{sensitivity}/\text{recall}=\;\frac{TP}{TP+FN} \end{array}$$
3. The specificity is the true positive cases recognition rate, which means the percentage of negative tuples correctly identified:
  (26) $$\begin{array}{l} \text{specificity}=\;\frac{TN}{FP+TN} \end{array}$$
4. The precision is a measure of accuracy, which means the percentage of tuples marked as positive that are positive:
  (27) $$\begin{array}{l} \text{precision}=\;\frac{TP}{TP+FP} \end{array}$$
5. *F*_1_ − *score* is the harmonic average of accuracy and recall rate, which means the recall rate is weighted once as much as the precision:
  (28) $$\begin{array}{l} F_{1}-\text{score}=\frac{2^{*}{rmprecision}^{*}\text{recall}}{\text{precision}+\text{recall}} \end{array}$$
6. Receiver operating characteristic (ROC) curves is a useful visual tool for comparing classifier models, which can provide objective and neutral advice regardless of cost/benefit when making decisions. The ROC curve shows the tradeoff between the true positive rate (TPR) and the false positive rate (FPR) for the classifier model. The increase in TPR comes at the expense of the increase in FPR:
  (29) $$\begin{array}{l} TPR=\;\frac{TP}{TP+FN} \end{array}$$
  (30) $$\begin{array}{l} FPR=\;\frac{FP}{FP+TN}\; \end{array}$$
  The Y-axis of the ROC curve represents TPR and the X-axis represents FPR, and the area under the ROC curve (*AUC*) is a measure of model accuracy:
  (31) $$\begin{array}{l} AUC=\;\frac{\left(TPR-FPR+1\right)}{2} \end{array}$$
7. Matthew's correlation coefficient (MCC) is a contingency matrix method (61). MCC can be used to calculate the Pearson product-moment correlation coefficient (62) between the actual value and the predicted value:
  (32) $$\begin{array}{l} MCC=\;\frac{TP^{*}TN-FP^{*}FN}{\sqrt{{\left(TP+FP\right)}^{*}{\left(TP+FN\right)}^{*}{\left(TN+FP\right)}^{*}\left(TN+FN\right)}} \end{array}$$

## Results

### Results of GRF, Joint Kinematics, and Joint Kinetics

**Figure 3:**
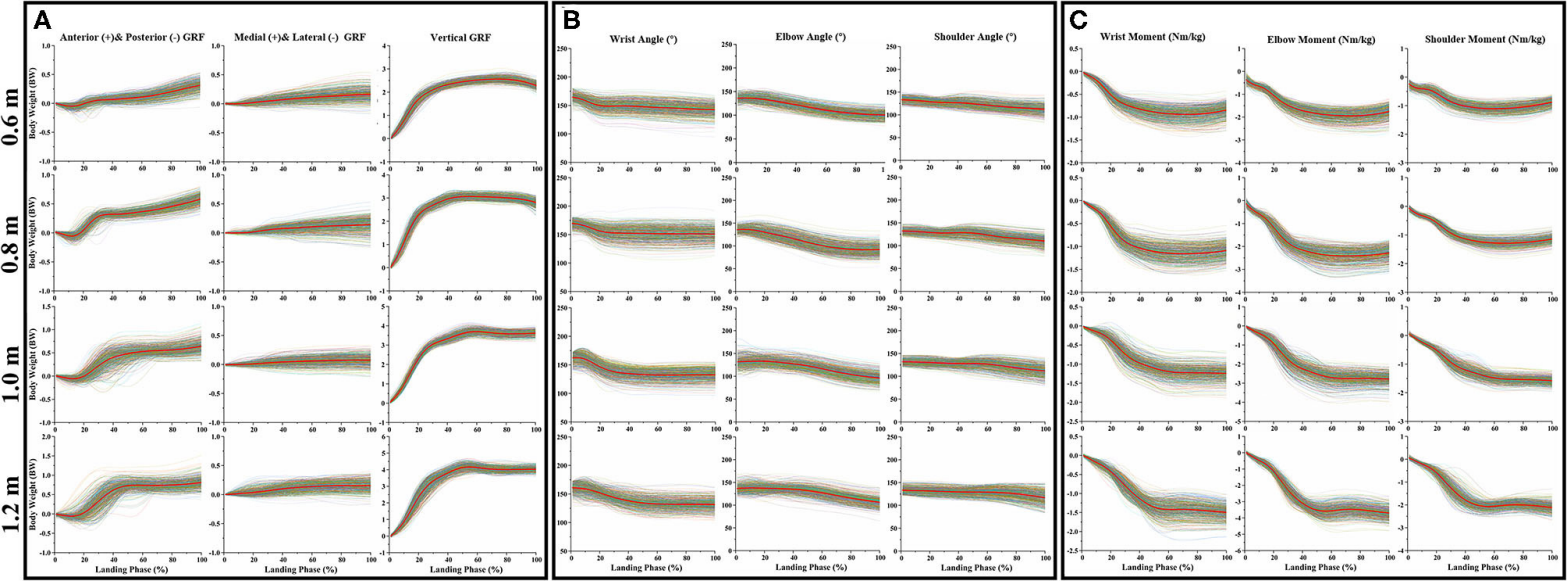
The data waveform curves of GRF, joint angle, and joint moment. **(A)** Illustrates the changes in GRF in three directions (anterior and posterior, medial and lateral, and vertical) during the cat landing phase from different heights. **(B)** Illustrates the changes of three joint angles (wrist, elbow, and shoulder) during the cat landing phase from different heights. **(C)** Illustrates the changes of three joint moments (wrist, elbow, and shoulder) during the cat landing phase from different heights. The colored data of each image represented 560 data sets recorded during the cat landing phase from different heights. The red line data of each image represents the average value of 560 data sets during the cat landing phase from different heights. On the left of each image, the scale displays the change of GRF/ angle/moment values. Zero to one hundred below each image presents a landing phase.

Figure 3A clearly shows the GRF dataset in three directions (anterior and posterior, medial and lateral, and vertical) during the cat landing phase from different heights (60, 80, 100, and 120 cm). Figure 3B clearly shows the joint kinematics (sagittal joint angle) dataset on three joint angles (wrist, elbow, and shoulder) during the cat landing phase from different heights (60, 80, 100, and 120 cm). Figure 3C clearly shows the joint kinetics (sagittal joint moment) dataset on three joint angles (wrist, elbow, and shoulder) during the cat landing phase from different heights (60, 80, 100, and 120 cm).

### Results of PCA

**Figure 4:**
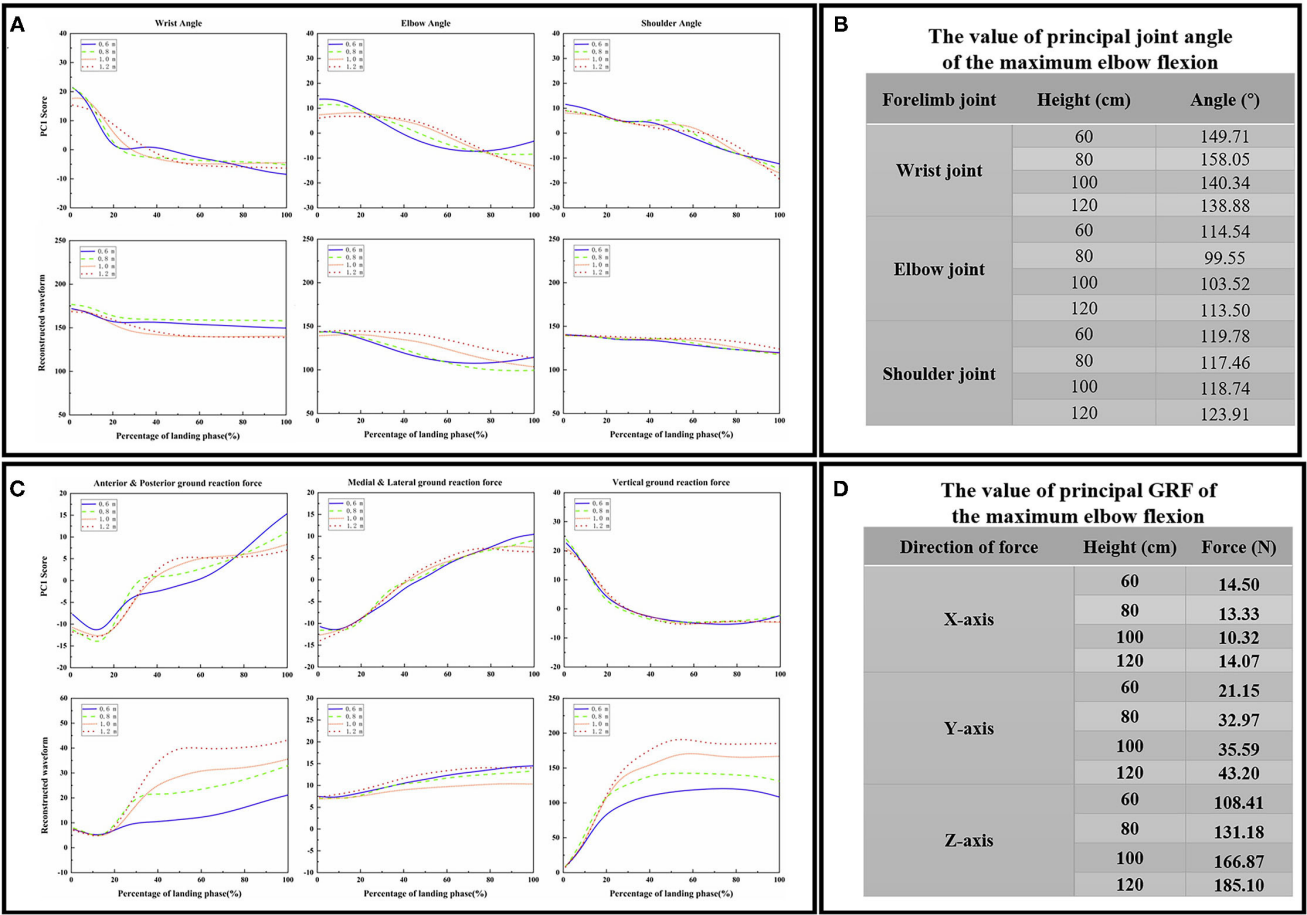
**(A)** The first PC scores *PC*_1_ for each landing height and each joint sagittal angle (wrist joint, elbow joint, and shoulder joint), the reconstructed waveform (principal joint angle) based on the first PC scores *PC*_1_. **(B)** The value of the principal joint angle of the maximum wrist flexion data point in each joint during landing from different heights. **(C)** The first PC scores *PC*_1_ for each landing height and each direction GRF (anterior and posterior, medial and lateral, and vertical), the reconstructed waveform (principal GRF) based on the first PC scores *PC*_1_. **(D)** The value of principal GRF of the maximum wrist flexion data point in each direction during landing from different heights.

The contribution rate of the first PC score for most variables was more than 90%, so the first PC was determined to reconstruct the waveform. The first PC score for each landing height and each joint flexion angle are shown in Figure 4A, the reconstructed waveform (principal joint angle) based on the first PC score are shown in Figure 4A. According to the principal joint angle, the joint angle value for each landing height for the maximum elbow flexion was extracted (the detailed values are shown in Figure 4B). The first PC score for each landing height and each direction GRF are shown in Figure 4C, the reconstructed waveform (principal GRF) based on the first PC score are shown in Figure 4C. According to the principal GRF, the GRF value for each landing height of the maximum elbow flexion was extracted (the detailed values are shown in Figure 4D). Finally, the joint angle (Figure 4B) and GRF (Figure 4D) data values of each landing height at the time point of the end of the landing phase (maximum elbow flexion) are extracted from the reconstructed waveform and substituted into the finite element model to investigate the stress distribution of the cat right forelimb bone.

### Results of FEA

#### Validity Verification of the Finite Element Model

For the validation of the cat's paw finite element model, the numerically predicted, and the experimentally obtained paw pressure distributions were compared. The paw pressure concentrated mainly on the metaphorical pad concerning the finite element or the experimental results. The numerically predicted contact area was ~57 cm^2^ in comparison to the experimentally obtained 54 cm^2^, which showed 5.6% higher over-prediction. The maximal pressure in the finite element model was located at the metaphorical pad in the measurement. The finite element model predicted a peak pressure of 0.25 MPa, while the experimental result, measured using a pressure platform, was 0.27 MPa, a difference of 8%. The results show that the numerically determined pressure distribution in the fore-left paw was in good agreement with experimental data, as shown in Figure 2E.

#### Stress Distribution of the Forelimb

**Figure 5:**
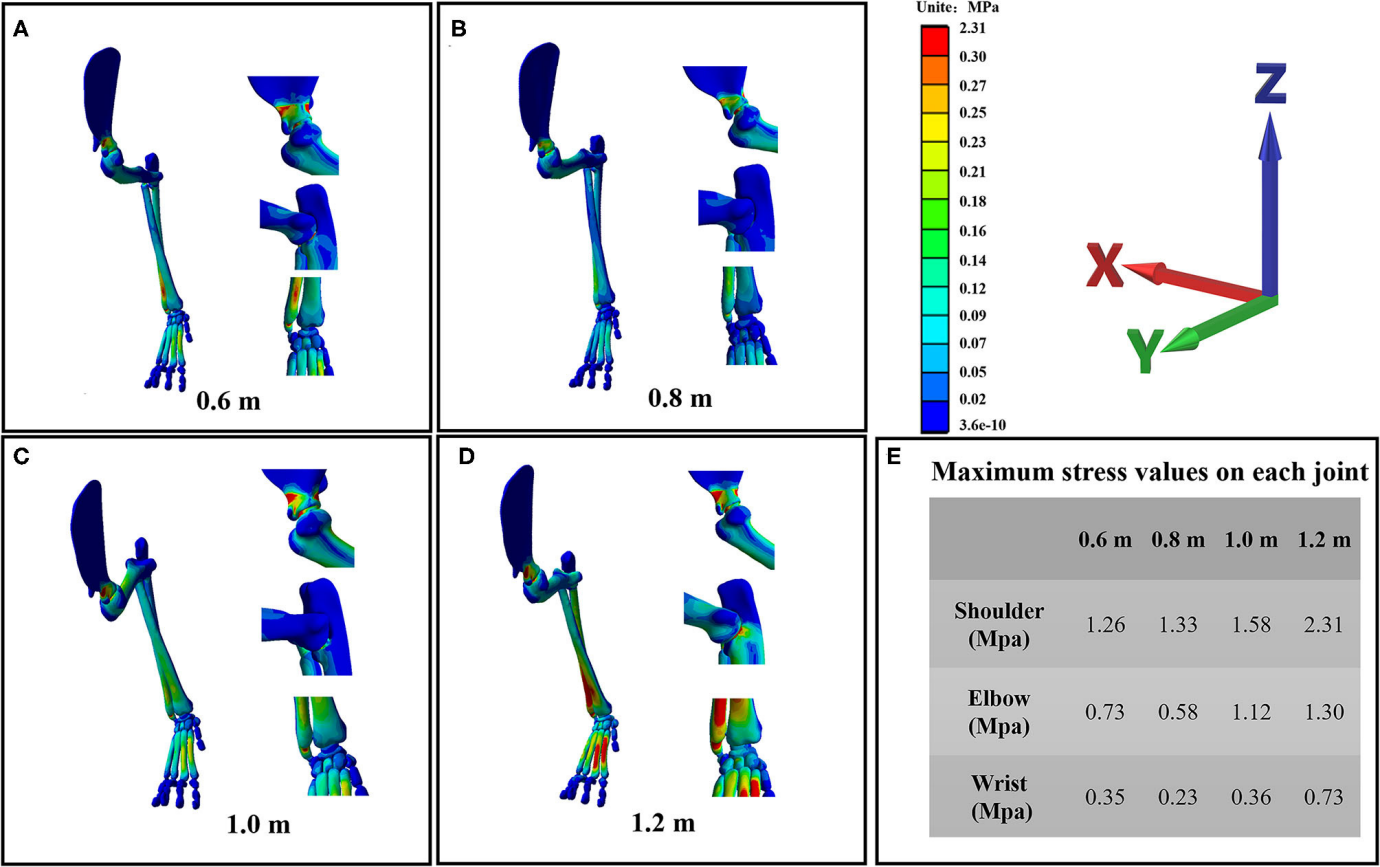
**(A–D)** are the stress distribution details of the wrist joint, elbow joint, and shoulder joint of the cat's right forelimb landing from four heights of 0.6, 0.8, 1, and 1.2 m, respectively. **(E)** The maximum stress value of each joint at each landing height.

The stress distribution of the forelimb as a result of falling from different heights is shown in Figure 5. From the stress distribution in the figure, it can be seen that the maximum stress of the forelimb is mainly concentrated in each joint when landing, indicating that the risk of joint injury is higher when the cat falls. However, the maximum stress of each joint does not simply increase with the increase of height, because the angle of each joint is also different when falling from different heights, so the stress change trend of each joint is also different. The maximum stress value of each joint is shown in Figure 5E. From the maximum stress value in the figure, we can see that when the cat hits the ground, the maximum stress value of the shoulder joint is the largest, followed by the elbow joint and the wrist joint, indicating that the shoulder joint is the most important buffer joint, followed by the elbow joint and wrist joint. This study found that the maximum stress of the elbow joint and wrist joint decreased compared with 0.6 m at 0.8 m height, while the angle of the elbow joint and shoulder joint was the smallest at 0.8 m height, and the angle of the wrist joint was the largest at 0.8 m height. In the case of increased height, the cat's forelimbs are bent to achieve a damping effect, but not all the joint bending damping effect is efficient, the wrist joint stress also has a downward trend, but its angle is the largest. Investigating the metacarpals of cats, it can be seen that in all metacarpals, stress is mainly concentrated in the second and third metacarpals.

### Performance of DNN Classification Models

For the matrices *M*_1_, there were 111 positive classes and 113 negative classes in the 224 test set samples extracted by a random function. Among them, 102 TP, 9 FN, 100 TN, and 13 FP were obtained by the DNN classifier. For the matrices *M*_2_, there were 114 positive classes and 110 negative classes in the 224 test set samples extracted by a random function. Among them, 108 TP, 6 FN, 107 TN, and 3 FP were obtained by the DNN classifier. For the matrices *M*_3_, there were 116 positive classes and 108 negative classes in the 224 test set samples extracted by a random function. Among them, 113 TP, 3 FN, 103 TN, and 5 FP were obtained by the DNN classifier.

**Figure 6:**
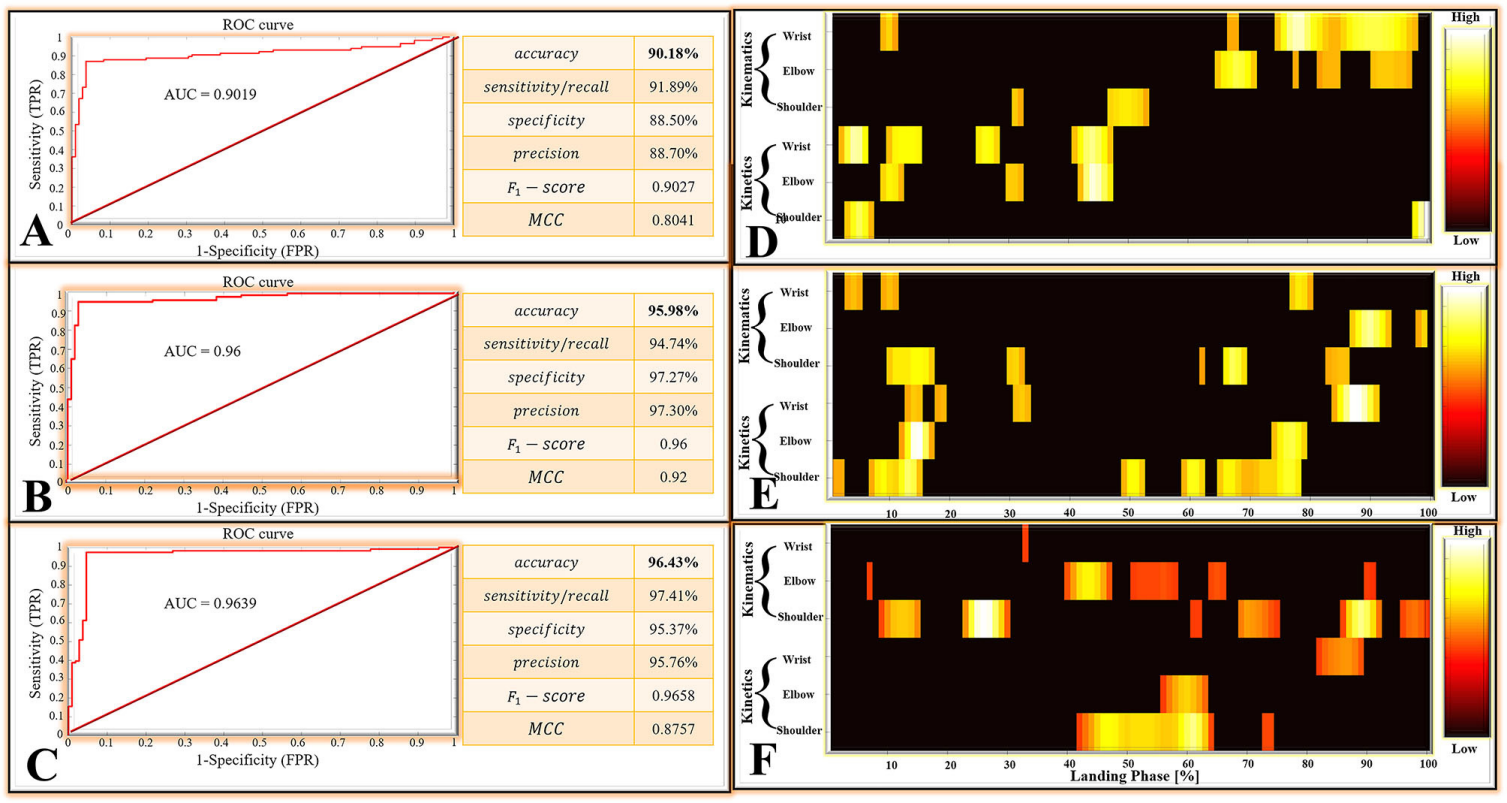
**(A)** The classifier performance results of the DNN models based on the matrices *M*_1_. **(B)** The classifier performance results of the DNN models are based on the matrices *M*_2_. **(C)** The classifier performance results of the DNN models are based on the matrices *M*_3_. ROC, Receiver Operating Characteristic; AUC, Area Under the ROC Curve; MCC, Matthews Correlation Coefficient; TPR, True Positive Rate; FPR, False Positive Rate. **(D–F)** Notable highly relevant variable during each joint (wrist, elbow, and shoulder) of kinematics (joint angle) and kinetics (joint moment). The top 100 variables with the highest correlation relevance. **(D–F)** are the result based on matrices *M*_1_, *M*_2_, and *M*_3_, respectively.

All classification performance parameters are presented in Figure 6. For the classifier of the DNN models based on the matrices *M*_1_, the model shows a lower accurate rate (accuracy rate: 90.18%) than the matrices *M*_2_ (accuracy rate: 95.98%) and matrices *M*_3_ (accuracy rate: 96.43%). At the same time, the classifier of the DNN models based on the matrices *M*_1_ also show the lower *F*_1_ − *score* (0.9027) and *MCC* (0.8041) than the matrices *M*_2_ (*F*_1_ − *score*: 0.96, *MCC*: 0.92) and the matrices *M*_3_ (*F*_1_ − *score*: 0.9658, *MCC*: 0.8757).

The ROC curves are shown in Figure 6, the ROC curves of the classifier of the DNN models based on the matrices *M*_2_ (Figure 6B) and the matrices *M*_3_ (Figure 6C) presented a good classification performance over the entire area. However, the ROC curves based on the matrices *M*_1_ (Figure 6A) show the worse classification performance during the about (0_*FPR*_−0.8_*FPR*_)*(0.88_*TPR*_−1_*TPR*_) area. The classifier of the DNN models based on the matrices *M*_1_ show the lower AUC (0.9019) than the matrices *M*_2_ (AUC: 0.96) and matrices *M*_3_ (AUC: 0.9639). Overall, the classifier of the DNN models based on the matrices *M*_1_ has a bad performance from the perspective of overall indicators.

### Results of LRP

For the results based on matrices *M*_1_, which compared the landing patterns between the cat landing from 60 cm platform and landing from 80 cm platform. For the LRP results based on matrices *M*_2_, which compared the landing patterns between the cat landing from 80 cm platform and landing from 100 cm platform. The LRP results based on matrices *M*_3_ compared the landing patterns between the cat landing from the 100 cm platform and the 120 cm platform.

**Figure 7:**
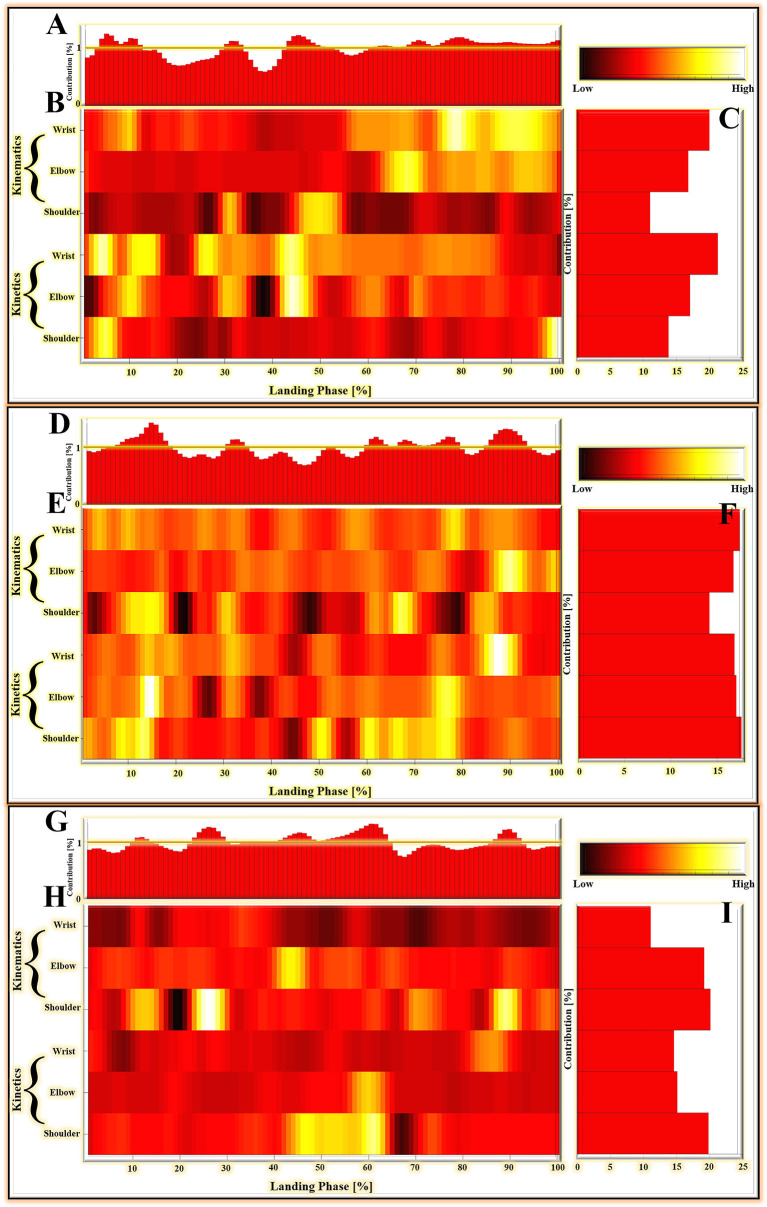
**(A–C)** The LPR results in the average absolute relevance score of every variable in the landing pattern based on matrices *M*_1_. **(D–F)** The LPR results in the average absolute relevance score of every variable in the landing pattern based on matrices *M*_2_. **(G–I)** The LPR results in the average absolute relevance score of every variable in the landing pattern based on matrices *M*_3_. **(A,D,G)** The relative contribution of variables during the overall landing phase (1–100%). **(B,E,H)** The detailed distribution of relevance score during each joint (wrist, elbow, and shoulder) of kinematics (joint angle) and kinetics (joint moment). The lighter colors mean high relevance variables; the darker colors mean low relevance variables. The model relied more on lighter-colored variables; the darker-colored variables had less relevance with correctly classified gait patterns. **(C,F,I)** The summed contribution of the relevance score of each joint (wrist, elbow, and shoulder) of kinematics (joint angle) and kinetics (joint moment) trajectories.

For the results based on matrices *M*_1_, *M*_2_, and *M*_3_, the relative contribution of variables during the overall cat landing phase are shown in Figures 7A,D,G, respectively. The variables recorded at every 1% of the landing phase interval are related to successfully matching the landing pattern. The detailed distribution of relevance score during each joint (wrist, elbow, and shoulder) of kinematics (joint angle) and kinetics (joint moment) are shown in Figures 7B,E,H (*M*_1_), (*M*_2_), and (*M*_3_). There were revealing findings contributing to the distribution of the variables on time points between the cat landing from different height platforms during the overall landing movement patterns. The summed contribution of the relevance score of each joint (wrist, elbow, and shoulder) of kinematics (joint angle) and kinetics (joint moment) trajectories are shown in Figures 7C,F,I (*M*_1_), (*M*_2_), (*M*_3_). For Figure 7C, the summed contribution of the relevance score of the wrist flexion angle, elbow flexion angle, shoulder flexion angle, wrist flexion moment, elbow flexion moment, shoulder flexion moment was 19.98, 16.81, 11.06, 21.25, 17.07, and 13.84%, respectively. For Figure 7F, the summed contribution of the relevance score of the wrist flexion angle, elbow flexion angle, shoulder flexion angle, wrist flexion moment, elbow flexion moment, shoulder flexion moment was 17.45, 16.78, 14.17, 16.88, 17.08, and 17.64%, respectively. For Figure 7I, the summed contribution of the relevance score of the wrist flexion angle, elbow flexion angle, shoulder flexion angle, wrist flexion moment, elbow flexion moment, shoulder flexion moment was 11.10, 19.18, 20.13, 14.63, 15.13, and 19.83% respectively.

There are 600 relevant variables in this study, including 6 trajectory variables, and each trajectory variable include 100 relevant variables (1%–100% landing phase). Notable highly relevant variables (the top 100 relevant variables with the highest correlation relevance) during the landing phase are shown in Figure 6. Figure 6D represents the results based on matrices *M*_1_: (1) For the wrist kinematics, there was a high relevance score in flexion angle during the 9–11, 67–68, and 75–98% landing phase; (2) For the elbow kinematics, there are high relevance scores in flexion angles during the 65–71, 78, 82–85, and 91–97% landing phase; (3) For the shoulder kinematics, there was a high relevance score in flexion angle during the 31–32, and 47–53% landing phase; (4) For the wrist kinetics, there was a high relevance score in flexion moment during the 2–6, 10–15, 25–28, and 41–47% landing phase; (5) For the elbow kinematics, there was a high relevance score in flexion moment during the 9–12, 30–32, and 42–47% landing phase; (6) For the shoulder kinematics, there were high relevance scores in flexion moment during the 3–7 and 98–100% landing phase. Figure 6E represents the result based on matrices *M*_2_: (1). For the wrist kinematics, there were high relevance scores in flexion angle during the 3–5, 9–11, and 77–80% landing phase; (2) For the elbow kinematics, there were high relevance scores in flexion angle during the 87–93 and 98–99% landing phase; (3) For the shoulder kinematics, there were high relevance scores in flexion angle during the 10–17, 30–32, 62, 66–69, and 83–86% landing phase; (4) For the wrist kinetics, there were high relevance scores in flexion moment during the 13–15, 18–19, 31–33, and 84–91% landing phase; (5) For the elbow kinematics, there were high relevance scores in flexion moment during the 12–17 and 74–79% landing phase; (6) For the shoulder kinematics, there was a high relevance score in flexion moment during the 1–2, 7–15, 49–52, 59–62, and 65–78% landing phase. Figure 6F represents the result based on matrices *M*_3_: (1) For the wrist kinematics, there were high relevance scores in flexion angle during the 33% landing phase; (2) For the elbow kinematics, there were high relevance scores in flexion angle during the 7, 40–47, 51–58, 64–66, and 90–91% landing phase; (3) For the shoulder kinematics, there was a high relevance score in flexion angle during the 9–15, 23–30, 61–62, 69–75, 86–92, and 96–100% landing phase; (4) For the wrist kinetics, there were high relevance scores in flexion moment during the 82–89% landing phase; (5) For the elbow kinematics, there were high relevance scores in flexion moment during the 56–63% landing phase; (6) For the shoulder kinematics, there were high relevance scores in flexion moment during the 42–64 and 73–74% landing phase.

## Discussion

This study was designed to investigate the biomechanical characteristics of a cat landing from different heights and provide new insights into bionic robot design based on the research results and the needs of bionic engineering. Specifically, the present work was to investigate the adaptive motion adjustment strategy of the cat's forelimb for each joint (wrist, elbow, and shoulder) during the landing phase using a machine learning algorithm and FEA. The present results suggest that as the cats' landing height gradually increases, the cat exhibits an adaptive movement adjustment strategy that gradually shifts from the distal joints (wrist joint) of the forelimbs to the proximal joints (shoulder joint) when responding to ground impact loads. Previous studies have shown that it is primarily the muscles of the limbs that act as dampers when cats land, dissipating impact and reducing damage (42, 63). Few studies have considered the role of joint coordination when cats land from a high place. The current results seem to provide new information and understanding of why when cats land from the high place they perform an excellent method of dissipating the impact of landing, thus protecting themselves from injury. The application of this intrinsic landing mechanism in the design of bionic robots is worth considering by related field researchers.

With the rapid development of bionic technology, bionic mobile robots have been widely used in military, scientific research, medical, aerospace, and many other fields. Among them, research into leg bionic mechanisms is very important to improve the movement ability of bionic robots (1). It is well-known that cats have significant advantages in terms of sports energy efficiency and sports flexibility compared with other animals. The cat's forelimbs play a leading role in free movement, while their hind limbs play a driving role (64). To compare a cat with a car, a cat's forelimbs may represent “the steering wheel” of the car and its hind limbs the “engine.” For landing motion, however, the cat's forelimbs play a crucial role in landing because they absorb most of the impact load (41, 42). In the current study, we investigated the biomechanical differences in cats landing from four different heights. The DNN classification model and LRP were used for pattern recognition and interpretability analysis of landing patterns at different heights. During the identification of landing modes with landing heights of 60 and 80 cm, we found that the contribution rates to the relevance score of the wrist joint were the greatest, reaching 41.23%, surpassing the elbow (33.88%) and shoulder (24.9%). During the identification of landing modes with landing heights of 80 and 100 cm, the contribution rates of the wrist (34.33%), elbow (33.86%), and shoulder joint (31.81%) were the same. When the landing heights were 100 and 120 cm, the contribution rates to the relevance score of the shoulder joint were the greatest, reaching 39.96%, surpassing the elbow (34.31%) and wrist (25.73%). With the increase in landing height (from 60 to 120 cm), the joint that contributed most to the landing pattern recognition gradually shifted from the distal joint (wrist joint) to the proximal joint (shoulder joint). This seems to be the adaptive movement adjustment that the cat demonstrates during the landing process. By autonomously adjusting the biomechanical mechanism of each joint landing at different heights, the cat can maximize the landing performance and reduce landing damage.

From a biomechanical standpoint, the paw pads, limb bones, and coordinated joints can operate as a multi-level (foot-level, limb-level, and joint-level) cushioning system when landing, effectively dissipating the shock (48). At the same time, the cat's limb muscles play a crucial role in the landing process (63). Previous studies have shown that the amplitude and duration of muscle activity during landing are autonomously modulated by the cat depending on the height of the landing (42, 63, 65). Therefore, the adaptive movement adjustment strategy exhibited by cats during landing may be the result of muscle mobilization and control. Why, with the gradual increase in landing height, does the main joint that bears the impact load gradually from the wrist joint move to the elbow joint, and up to the shoulder, but not toward the wrist joint? It could be the cat's unique landing mechanism, or maybe it is more “economical” with the cat's landing mechanism. Of course, these speculations need to be further confirmed, and the specific causes also need to be further investigated.

When a bionic robot lands, it needs to face many problems such as contact collision and friction with the ground. Contact impact with the ground is a complex phenomenon, as factors such as material properties and contact surfaces need to be considered (44, 66). The impact of the contact between the mechanical leg and the ground will cause the vibration of the bionic robot, thus affecting its stability, service life, and control accuracy (67–69). From the view of FEA results, when the cat landed at different heights, the maximum stress of each joint appeared in the styloid process of the ulna, the coronal process of the ulna, and the neck of the scapula. The wrist and elbow joints are similar to the easily fractured parts of humans, but the easily fractured parts of shoulder joints are different from those of humans. The most prone parts of the human shoulder joint are the acromion and the acromial end of the clavicle, followed by the neck of the scapula. This is mainly due to structural differences (70). There is a great difference in the function of the cat's forelimb and the human upper limb. The cat's forelimb acts as a buffer when landing. With the evolution of biology, the cat's acromion and collarbone shorten, and this structure does not have direct comparisons with humans. As a result, the risk of fracture of the acromion is greatly reduced. The position of the maximum stress distribution is mainly distributed in the narrower parts at the two ends of the bone. In the design of bionic robots, the hardness of the joint should be strengthened to reduce machine wear and tear, thus affecting the control accuracy and reducing service life.

In addition to the material properties of the machine and the coordination mechanism of each joint, the posture of the bionic robot during landing is also the key to a good cushioning mechanism (71). At the same time, the bionic robot can control body posture to achieve a stable motion state in the air attitude adjustment stage, which can also provide a basis for a good landing (72). With the increase of height, the GRF increases, but the maximum stress of each joint does not increase, which was related to the angle of the cat's forelimbs. It can be seen from the results that the cat has the largest angle of the wrist and the smallest angle of shoulder and elbow when landing at a height of 0.8 m, and the body shows a posture of leaning forward and a low center of gravity. When landing at this height, the stress of the elbow joint and wrist joint is lower than that of other joints, which is caused by low height and small GRF and the low center of gravity and forward posture of the body. The relationship between the specific posture and the stress of each joint needs to be further explored. Falling with this kind of posture will reduce the stress of the lower limb joints to a certain extent, which has some implications for the falling posture of the bionic robots.

Over time, tetrapods have evolved many unique biological structures that help them adapt to a variety of environments and terrains. The design of bionic robots based on these animals with excellent locomotion ability needs to consider various factors. From the results of the present study, when cats land from lower heights (60 and 80 cm), the adaptive movement adjustment strategy makes the wrist joint the main joint that bears the impact load. With the gradual increase in landing height (100 and 120 cm), the main joint that bears the impact load gradually moves to the elbow joint, and up to the shoulder. Further research is needed to understand exactly why cats exhibit this landing mechanism. However, there is no doubt that this adaptive movement adjustment strategy allows the cat to cushion the impact load when landing, improve landing stability, and reduce landing damage. Therefore, when designing bionic robots, we suggest that the coordination mechanism of each joint should be adjusted intelligently according to the force at the bottom of the mechanical leg so that the robot can better buffer the impact load during the landing process. Specifically, with the increase of the force at the bottom of the mechanical leg, the main joint bearing the impact load gradually shifts from the distal joint to the proximal joint. We also found that the position of the maximum stress distribution is mainly distributed in the narrower parts of the two ends of the bone. Therefore, it is necessary to strengthen the hardness of materials around the center of each joint of the bionic robot leg. Finally, lowering the center of gravity of the robot during the landing process and keeping the posture forward as much as possible are also important mechanisms to reduce machine wear and improve the accuracy of robot operation. In conclusion, this study can directly provide a biomechanical theoretical basis and technical support for the innovative design and development of bionic robots with high energy efficiency motion characteristics and has important scientific significance.

There are some limitations in the present study. (1) The results of this study could be influenced by different varieties, genders, ages, and weights of the cats. In our ongoing study, we will expand our test sample to validate the results of this study. (2) We only built one FEA model to analyze the data, and the model won't be representative of all the features of the cat. We will engage more FEA models of cats during the next study to avoid results that might be affected by individual differences. (3) The finite element model in this study is only a simplified model, which simplifies the material properties of bones and ligaments to linear materials, and does not involve the internal and external forces of forepaw muscles and tendons. The ligaments were modeled by a straight node-to-node link element, and the specific variation in ligament positioning in the standing phase was not considered. Specific changes in the position of the ligament in the falling stage were not taken into consideration, so the model needs further optimization. (4) In this study, the FEA only explored the stress distribution at the maximum elbow flexion, and subsequent studies should also investigate other time points of the landing phase.

## Data Availability Statement

The raw data supporting the conclusions of this article will be made available by the authors, without undue reservation.

## Ethics Statement

The animal study was reviewed and approved by Animal Care and Use Ethics Committee of Ningbo University. Written informed consent was obtained from the owners for the participation of their animals in this study.

## Author Contributions

All authors have made substantial contributions to the manuscript. DX, HZ, and YG were responsible for the conception and design of the study. DX, XJ, QZ, SL, JB, and YG were responsible for data acquisition and data processing. HZ, XJ, QZ, and JB were responsible for data analysis and interpretation and drafting the article. DX and YG revised the manuscript critically. All authors read, provided feedback, and approved the submitted version.

## Funding

This study was sponsored by the National Natural Science Foundation of China (No. 81772423), the Key Project of the National Social Science Foundation of China (19ZDA352), Key R&D Program of Zhejiang Province China (2021C03130), and K. C. Wong Magna Fund in Ningbo University.

## Conflict of Interest

The authors declare that the research was conducted in the absence of any commercial or financial relationships that could be construed as a potential conflict of interest.

## Publisher's Note

All claims expressed in this article are solely those of the authors and do not necessarily represent those of their affiliated organizations, or those of the publisher, the editors and the reviewers. Any product that may be evaluated in this article, or claim that may be made by its manufacturer, is not guaranteed or endorsed by the publisher.
